# Putting the social back into sociotechnical: Case studies of co-design in digital health

**DOI:** 10.1093/jamia/ocaa197

**Published:** 2020-10-12

**Authors:** Chrysanthi Papoutsi, Joseph Wherton, Sara Shaw, Clare Morrison, Trisha Greenhalgh

**Affiliations:** 1 Nuffield Department of Primary Care Health Sciences, Medical Sciences Division, University of Oxford, Oxford, United Kingdom; 2 Technology Enabled Care Programme, Scottish Government, Edinburgh, United Kingdom

**Keywords:** co-design, sociotechnical theory. video consultations, telehealth

## Abstract

**Objective:**

We sought to examine co-design in 3 contrasting case studies of technology-supported change in health care and explain its role in influencing project success.

**Materials and Methods:**

Longitudinal case studies of a seizure detection and reporting technology for epilepsy (Southern England, 2018-2019), a telehealth service for heart failure (7 UK sites, 2016-2018), and a remote video consultation service (Scotland-wide, 2019-2020). We carried out interviews with 158 participants and collected more than 200 pages of field notes from observations. Within- and cross-case analysis was informed by sociotechnical theory.

**Results:**

In the epilepsy case, co-design prioritized patient-facing features and focused closely around a specific clinic, which led to challenges with sustainability and mainstreaming. In the heart failure case, patient-focused co-design produced an accessible and usable patient portal but resulted in variation in uptake between clinical sites. Successful scale-up of video consultations was explained by a co-design process involving not only the technical interface, but also careful reshaping of work practices.

**Discussion:**

A shift is needed from co-designing with technology users to co-designing with patients as service users, and with healthcare staff as professionals*.* Good co-design needs to involve users, including those who engage with the technology-supported service bothdirectly and indirectly. It requires sensitivity to emergence and unpredictability in complex systems. Healthcare staff need to be supported to accommodate iterative change in the service. Adequate resourcing and infrastructures for systems-focused co-design are essential.

**Conclusions:**

If co-design focuses narrowly on the technology, opportunities will be missed to coevolve technologies alongside clinical practices and organizational routines.

## INTRODUCTION

### Background and Significance

It is widely accepted that health technologies should be co-designed to make them more acceptable and usable to staff and patients.^1–4^ Yet, co-design has different meanings; for some, it means testing technologies in development and making changes based on user feedback^5^; for others, it denotes in-depth, deliberative engagement with users’ routines and priorities, alongside mutual adaptation of technologies within sociotechnical practices.^6^^,^^7^

Sociotechnical design has its roots in the work of the Tavistock Institute, around how socially and culturally shaped human behavior profoundly influences technology use, and how technologies, in turn, shape and constrain human behavior.^8–10^ This approach also underpins human factors engineering that seeks to optimize task performance by accounting for human capabilities and limitations in complex environments.^11^ In taking forward these lessons, computer-supported cooperative work emphasizes articulations (or “workarounds”) that users develop to overcome the “brittleness” of digital technologies; design is seen as ongoing and social as well as technical, situated, and adaptive.^12–14^ This foregrounds the role of ethnography, not only to improve task performance, but also to understand situated users’ needs.^15^ From an organizational perspective, Braithwaite et al^16^ draw on similar concepts to distinguish “work-as-imagined” (an idealized and highly standardized version of a practice or routine) from “work-as-done” (how a staff member actually performs the work under real-world pressures). 

Co-design has the potential to bring into focus sociotechnical aspects of clinical work and patient self-care, and lead to user-oriented change in health informatics and service improvement.^17^ Three key sociotechnical approaches to technology design have recently been described in the medical informatics literature: community-based participatory research, in which projects rely on strong community partnership and stakeholder engagement across all stages; participatory design, in which collaboration with those identified as end users becomes more focused and purposive; and user-centered design, in which end-user input becomes limited around specific design tasks.^1^^,^^18^ Other approaches used in health services research include experience-based co-design and service co-production.^19^^,^^20^

Co-design becomes more productive when viewed as an iterative process of development, value creation, and knowledge generation.^21^ However, significant effort is required to build meaningful and sustainable community partnerships over time and to manage diverse interests, shifting priorities, and communication needs between partners, including the involvement of “vulnerable” groups.^1^^,^^22^^,^^23^ Owing to these challenges, in practice, many digital health implementations remain project-oriented and firmly focused on the technology itself, engaging only selectively with the “social.”^24^Co-design becomes limited to optimizing technical characteristics prior to a randomized controlled trial (RCT) to test the technology’s “impact” on a predefined variable. Such studies are often inherently deterministic and do not, either philosophically or empirically, address the ongoing adaptation of a complex system characterized by unpredictability and emergence.^25^

In this article, we take a broad view of co-design to refer to the processes by which technology becomes recursively designed and adapted within human and organizational practices—including how these practices become shaped in the context of technology co-design and implementation. We are also interested in how heterogeneous representations of intended users become articulated in co-design processes, particularly focusing on the co-constitution between users, design practices, and materialities.^26–28^ This framing, based on technology design, adoption, and use as adaptive, sociomaterial practices,^29^ explicitly recognizes involvement of people in interpreting and fitting technologies in care practices, and attends to technologies as institutionally sanctioned and supported.

### Objective

We sought to explore co-design in 3 case studies—a seizure detection and reporting technology for epilepsy, a remote monitoring service for heart failure, and a video consultation service for people living in remote areas. All 3 cases aimed to introduce a new service, for which technology was a crucial (but not the only) component. Our hypothesis was that successful uptake of the service (ie, in a routine and sustainable way) would depend on initial co-design of the technology and ongoing co-design of sociotechnical practices in healthcare organizations (and likely patients’ routines).

Our empirical research questions were the following: (1) What kinds of planned and emergent co-design occurred in each case? (2) How were different groups involved? (3) What practices and work routines were *not* included in the co-design effort? and (4) What were the challenges to the co-design efforts? Our overarching question was the following: what generalizable lessons can we draw from these case studies about how to improve co-design in complex, technology-supported change?

## MATERIALS AND METHODS

### Empirical approach and data sources

The study consisted of 3 longitudinal case studies introduced in Box 1. We used an interpretive case study approach to generate a rich understanding of each technology project, also drawing on a set of reporting principles currently in development by our team.^30^ We selected our 3 cases as they provided contrasting data across specific dimensions: (1) different “organizing visions” for technology projects (eg, increasing efficiency, reducing variation or driving quality improvement), (2) different starting points for co-design (eg, focusing on the technology or the service), (3) different contexts for carrying out co-design (eg, within or outside the clinic), and (4) different stages in technology development. Our focus on co-design formed part of larger evaluations, with study findings for 2 of the projects published elsewhere (unpublished data, C. Papoutsi, PhD et al, 2020).^31^Table 1 includes a full account of our data sources. Research participants (N = 158) included patients, caregivers, frontline clinical and administrative staff, information technology (IT) professionals from public and commercial organizations, and policymakers. Semi-structured interviews used topic prompts (available from authors) that were customized to the type of interviewee and adapted as data collection progressed. Most interviews were carried out individually, although a small number involved carers or family members; discussions were audiotaped with consent and professionally transcribed. To develop our case narratives and guide further data collection, we also collected project documents (eg, protocols, minutes of meetings, technical specification), which we analyzed for relevant data (eg, rationale for co-design, changes to technical specifications following patient feedback, processes followed). We supplemented this data with ethnographic observations primarily in clinical settings, focusing on consultations and other aspects of patient care in which technology was used, as well as on organizational practices. These observations went beyond pure mechanistic description of the processes followed, to place the meaning of our observations into context and help problematize taken-for-granted understandings (including our own), consistent with Diana Forsythe’s approach.^32^^,^^33^ We do not suggest that we carried out full ethnographies in the anthropological sense or that ethnography is our overarching research design in each of the 3 studies. Rather, we highlight how we retained ethnographic sensibilities in our engagement with sites, including an understanding of the “field” and its social practices as phenomena to be interpreted, rather than existing out there to be observed and captured.^33^

**Table 1.** Summary of data sources

	Case 1: Seizure detection and reporting in epilepsy	Case 2: Remote monitoring for heart failure	Case 3: Video consultations in remote areas
Duration of data collection	2017-2018 (18 mo)	2016-2018 (26 mo)	2019-2020 (9 mo)
Interviews	29 participants total Service users (n = 24): 13 patients and 11 relatives Staff (n = 5): 1 consultant neurologist, 1 specialist epilepsy nurse, 1 professional carer, and 2 technology professionals. Additional email and phone discussions with patients.	51 participants total Service users (n = 28): 25 patients and 3 relatives (including 1 discussion group) Staff (n = 23): 4 cardiologists in the RCT, 4 research nurses, 10 heart failure specialist nurses (2 hospital, 8 community), 1 nonclinical researcher, 1 administrator, 1 trial manager, 1 general practitioner, and 1 bioengineer.	78 participants total Service users (n = 16): 12 patients and 4 relatives Staff (n = 49): 12 doctors, 14 nurses, 6 allied professions, 2 healthcare support workers, 4 clinician-managers, 8 nonclinical managers, and 3 IT managers. National stakeholders (n = 13): 5 national policymakers, 4 national program managers, 3 systems engineers and national technical support, and 1 technology industry.
Field notes and observations	Ethnographic observation in clinical consultations (n = 26 first and follow-up consultant and nurse appointments) Ethnographic observation during IT “troubleshooting” with patients Observation of patients using the wearable and app	Examination of the technologies (patient peripherals and tablet, central dashboard) Observation of patients using the tablet and central support nurse using the dashboard 5 meetings of the trial implementation team Significant event review meeting relating to an alleged missed deterioration	Examination of the technologies (partly by using the platform to conduct video calls and interviews) “Walkthrough” of both face-to-face and virtual arrivals at reception desk and transfer to waiting room at hub and peripheral sites Observation of patients having video consultations (n = 6)
Documents	Project documentation, including details of the technology Published articles and abstracts, reporting pilot findings	Trial protocol Minutes of meetings and approximately 60 emails with trial team Published articles reporting on trial findings	Technical documentation National Technology Enabled Care policies Implementation protocols and guides for video consultations

IT: information technology; RCT: randomized controlled trial.

### Data analysis

Data were imported into NVivo 12 (QSR International, Melbourne, Australia) (cases 1 and 2) or Microsoft Excel (Microsoft Corporation, Redmond, WA) (case 3) to support data management. For each case, we produced an overarching narrative that we progressively refined over time, adding each new data item (from interviews, field notes, or documents) to an increasingly nuanced account developed collaboratively in team meetings (see Box 1 for a short version of our case narratives).^34^ We then returned iteratively to our dataset for each case to look specifically for material on co-design—that is, data on the processes by which technology became co-shaped through different kinds of interactions with end users and other stakeholders, either in an ad hoc or in a planned manner throughout the system life-cycle.

We carried out conceptual coding across data sources to understand how co-design was approached (eg, formal or informal methods used, stakeholder groups included (or not), co-design aspects missing or neglected) (see Table 2). We extended initial deductive coding not only with new themes emerging from within-case analysis, but also by comparing and contrasting across cases. Theoretically, we drew on a sociotechnical lens for the analysis and interpretation of our data, as explained in the Background and Significance section. For example, we examined the theoretical assumptions underpinning co-design, and the extent to which it was viewed as a technical endeavor or as ongoing sociotechnical process taking in account values, routines, and authority structures. Our approach to theory-driven research goes beyond prescriptive and compartmentalized application of theoretical concepts; instead, we were interested in interrogating the assumptions by which programs were developed and employed our theoretical orientation in a way that surfaced the nuances of different contexts.^25^^,^^35^^,^^36^

**Table 2.** Overview of 3 cases

	Case 1: Seizure detection and reporting in epilepsy	Case 2: Remote monitoring for heart failure	Case 3: Video consultations for remote patients
Description of sites	Specialist epilepsy service with 1 neurology consultant and 2 specialist nurses covering a county in the Southwest of England (population around 400 000). The clinical team were primarily based in 1 NHS Foundation hospital but provided clinics in other secondary and community care organizations, including home visits.	7 cardiology services across the United Kingdom, all participating in a telehealth trial: 1 central “hub” site with cardiology professor and support nurse 6 “spoke” sites, each with hospital-based heart failure service with cardiologist, research nurse and/or specialist nurse, plus (in some sites) a community cardiology service	6 sites (from a total of 20 participating in a national technology-enabled care program across Scotland): 1 major teaching hospital that served as a “hub” to which patients connected for most specialist video consultations 3 remote hospitals which served as “spokes” from which patients connected to the teaching hospital and also as “hubs” offering basic secondary care consultations 2 GP practices
Sector	Secondary care	Primary and secondary care (interface)	Mainly secondary care
Project leadership	Clinical	Clinical academic	Scottish government, with local leadership in NHS boards
What was the organizing vision for the technology?	Rationalizing epilepsy care using state-of-the-art technologies; improving service efficiency by supporting self-management.	Using technology to disseminate evidence-based standards and guidelines; (thereby) reducing variation in care.	Quality improvement; improving access to health care by patients living in remote areas; reducing inequalities; addressing climate emergency targets by reducing travel.
Who were the technology’s [assumed] users?	Patients, carers, neurologists, and specialist nurses	Patients, carers, cardiologists, and research nurses (and some community clinicians)	Patients, specialist clinicians in central sites; support staff in remote sites
What was the starting point for co-design?	Mainly, the technology	Mainly, the technology	Mainly, the service
When did co-design occur and what was its purpose?	At early stages, to increase the technology’s acceptability to a relatively small group of patients in the pilot, and to establish back-end processes within the clinical service.	Prior to implementation in a RCT, to optimize the technology’s user interface. Some changes continued as part of the trial.	Beginning with a fairly mature technology, ongoing iteration of both the technology and a range of clinical services, with the aim of spreading and sustaining a service.
Examples of co-designed changes in this study	The login process on the app was simplified following patient feedback. Battery life for the smartwatch was too short and unreliable, which led to replacement of some devices.	Patients in the co-design workshop asked for layered access to data rather than directly illustrating detailed time graphs.	Patient feedback helped shape the Web portal from one that required self-navigation to one in which patients were supported by a real, human receptionist (as in a face-to-face clinic). Patients also chose the name of the service.

GP: general practitioner; NHS: National Health Service; RCT: randomized controlled trial.

## RESULTS

In this section, we first provide an overview of co-design efforts in each technology project: how co-design took place; what groups became involved; and what practices and work routines were included (or not) (also see Table 2 for a summary). This then leads to the analysis of 3 key challenges that played out differently across the cases.

**Case 1: Co-design to “modernize” epilepsy care**

The epilepsy project began with a workshop to elicit patient preferences for recording seizures; the session involved a small group of patients, along with the epilepsy specialists and IT professionals leading this project. Co-design focused mainly on ensuring that seizure detection and reporting technologies captured key information needed for clinical decisions (eg, seizure frequency) and that patients found the seizure app easy to log into and use (eg, making dropdown menus intuitive and meaningful). The commercial partners used agile methods and their “screen designer*”* tool for rapid modifications. In designing iteratively, they sought to balance patients’ priorities (eg, format of dropdown menus) with the information needs of the clinical team and with data analytics requirements for seizure detection. Following the initial patient workshop, IT partners spent time not only with clinicians and patients in clinical consultations, but also IT troubleshooting (rather than formal co-design meetings) to better understand how the service worked and how the technology could be adapted:

*“[…] they've all spent time with us. Which I think has given them a bit more understanding about epilepsy, but also about what we do. And just feeding back to them the practicalities.”* (Interview 21, specialist nurse)

However, technical challenges loomed large (eg, short battery life), as the materiality of the wearable device limited what the co-design could achieve (with a fragile wristband unsuitable for some patients with violent motor seizures), and additional development became difficult when external funding ended. With only a relatively small group of patients and enthusiastic clinicians contributing to the process, it was difficult to co-shape the technology for a wider set of complex health and care needs and to implement at scale. Priority was given to how the technology could be used to collect clinical data with less emphasis placed on how patient needs might diverge in ways that would affect continued use.

**Case 2: Co-design to “close the feedback loop” in heart failure patients**

The heart failure project organized a full-day co-design workshop with 15 heart failure patients and their caregivers, mainly focused on guiding selection of commercially available, tablet devices and refining the user interface.

*“So, the first set of questions was about the [pilot interface] designs […] which one is more user friendly, whether they think the new functionalities are useful to them; are they easy to use, would they want to use it. But also very small things like is the font size good enough; are the colours easy to differentiate, are the icons meaningful to you and that was really on the design phase.”* (Interview 4, project manager)

The resulting system was then piloted with 52 patients and their caregivers, focusing on patient usability and functionality improvements (eg, data visualization, automatic updates, instructions for taking measurements and synchronization errors), taking account of cognitive, digital literacy, and general fitness issues in this patient population. Some of the usability evaluation was done remotely through automated collection of user interactions; we also used follow-up home visits to understand how patients made sense of and appropriated the technology and its protocol in their routines.^37^ This process led to several improvements to the patient interface to optimize appeal and usability (eg, layered access to data). There had been less emphasis on co-design with staff, oriented to the working relationships and practices that the technology would support, beyond algorithm modifications for flagging patients at risk of deterioration and adaptations to facilitate trial operationalization. Once the RCT had begun, modifications to the technology continued, though they became more reactive and problem-focused.

**Case 3: Co-design to make services more accessible through video**

The technology selected for a major expansion of video consultations in Scotland had already been in use for video consultations in Australia and was deemed to have been co-designed to better reflect the workflows of a medical clinic, compared with other corporate video conferencing solutions commonly used for remote consultations.^38^ This feature had been foregrounded because of growing evidence that video consultations fail primarily not because the video connection itself is problematic, but rather because such consultations generate considerable work elsewhere in the system due to poor alignment with administrative routines.^39^

Having selected a bespoke technology, further co-design within Scotland was focused on “getting the service to work” and delivering tangible and scalable improvements in practice. The process of service co-design involved public information sessions, focus group workshops and mock video consultations with patients, telephone interviews and questionnaires—followed by iterative testing and learning cycles.^40^^,^^41^ The team actively maintained a balance between formal, participatory co-design opportunities using established tools, such as process mapping, and more flexible and opportunistic methods. The involvement of patients in service co-design (as opposed to just the technology) provided opportunities to develop and sustain a shared vision. Patients came up with the name of the video consultations service, suggested open testing sessions to increase their confidence with video consulting, and proposed that a receptionist would greet patients and transfer them into the relevant virtual waiting area for their appointment as in the face-to-dace service, rather than patients having to navigate different links for their different appointments^40^^,^^41^:

*“Patients said, why can’t we have a much simpler model and go via a receptionist […they] wanted that ‘normality,’ something they were used to.”* (Interview 1b, project lead)

*“I’ve been to [the spoke clinic] 3 or 4 times and I would say there is not much they can improve on it…. The only thing I would suggest—on a light note—is to change the music. While you are waiting, the music comes on. They could do with changing it now and again.”* (Interview, hematology patient)

Co-design also resulted in a series of technical, infrastructural, and organizational changes through close working with clinical and nonclinical staff, including regular feedback by email (“we sent her [the project lead] 3-page emails with long lists of things to change, and she kept improving things” [Interview 2, psychiatrist]) and even reaching out to local planners in council meetings. This case illustrates a very different kind of co-design—one in which a national policy priority is being pursued through the explicit use of formal quality improvement methods with a wide range of patients, clinical, and nonclinical staff as active partners. The technological platform, though essential, is seen as a means to a policy end, rather than an end in itself.

### Cross-case findings: what were the challenges to co-design efforts?

We found 3 key challenges to co-design efforts: (1) identifying and engaging different user groups as prospective users of the technology, either directly or indirectly; (2) balancing the tension between co-design as a separate, preliminary phase vs co-design as an ongoing, perhaps never-ending, process; and (3) ensuring appropriate resources, and human and technological infrastructures, are in place to support meaningful co-design practices.

#### Who are the prospective “users” that the co-design process serves?

As described previously, all projects engaged in iterative, participatory work with patients and frontline staff. However, their different starting points meant that different user representations were prioritized in co-design practices. Two projects (epilepsy and heart failure) focused more strongly on technology co-design with the aspiration that, if proven usable and effective, the technology would subsequently bring value to wider service redesign; patients were seen primarily as “technology users.” The third project (video consultations) had a different starting point, partly because the technology was more mature and had already been implemented elsewhere; the focus of the Scottish project was service reconfiguration, with refinements to the technology targeted around immediate service provision needs; therefore, working both with healthcare staff as “professionals” and with patients as “services users.” These different starting points had implications for defining who counts as a user and how they would be involved in co-design.

In the epilepsy case, strong clinical input was deemed necessary to guide patient engagement in technology co-design. This was due partly to the complexity of the condition, encompassing more than 40 syndromes with a range of different characteristics and comorbidities, and also related to the clinical challenge of distinguishing between epileptic and nonepileptic (assumed psychogenic) seizures. Clinicians decided that patients experiencing the latter would not be included in user representations for the project, as they would be submitting the “wrong” data for seizure detection algorithms. In exploring user representations through different co-design practices, technology providers had to find a balance between clinical needs and patient priorities “to maintain engagement from patients” (epilepsy case, interview 20, IT professionals). Tensions emerged when clinicians started having discussions around service reconfiguration with patients who had been using the technology effectively for some time. One of the more enthusiastic technology users who saw the technology as enhancing rather than replacing her usual care was disappointed when the consultant suggested a potential reduction in clinic appointments: “happy to do Skype and the reports [on the remote monitoring app] but I do like the face-to-face. Sorry” (epilepsy case, observation in clinic).

In the heart failure case, pretrial co-design primarily included patients and their caregivers, and focused on ensuring that tasks could be completed as required in preparation for the complex intervention to be tested in the trial. Importantly, clinicians beyond the trial team (cardiologists and research nurses) were not envisaged as active users when the technology (and the trial) was being designed—though it was imagined that they would implement recommendations based on risk prediction from the system. The technology’s designers had not anticipated that this would generate professional conflict, as the distant, academic authority (generic national guidelines) inscribed in the software came up against the “personal knowledge” and clinical accountability of the patient’s family physician or community heart failure nurse. Because the emphasis had been on technology co-design, rather than on service co-production, there was limited scope for developing a shared vision for the service, mutually shaping work practices and co-producing an appropriate place for technology-guided medicines optimization in heart failure care. A somewhat idealized and generic feedback loop to optimize clinical treatment had been imagined during the design phase, which was not always feasible to implement locally as a standard care pathway due to contextual differences across sites including caseload, levels of specialist skills and resources in both hospital and community teams, and the historically determined distribution of work between different professional groups in managing heart failure patients.

Despite efforts, the projects did not always manage to engage the number or diversity of participants they were looking for. Only small and relatively homogeneous groups expressed interest in co-design. Beyond access and scheduling challenges, it also seemed that patients did not always imagine themselves as potential users of these technologies or recognize what role they could play in the co-design process. To maximize the value of early co-design in the heart failure case, it became preferable to “just have the maximum variation and mainly pick people who are sort of very vocal, people who just come with extra wishes during the course of the study and very critical” (interview 5, cardiology consultant). Although this variation might have been helpful from the perspective of designing technology features, it likely introduced assumptions around how this technology might be embedded in the service.

In the video consultations case, the question of who counts as user became more easily answerable in the context of service provision: “We had people who I’m sure wouldn’t have come out to a group but would come to their appointment” (video consultations case, interview 1b, project lead). This allowed a shift in co-design thinking toward working with patients as active partners in service co-production, rather than purely as prospective technology users outside the service. Explicit focus on staff and patients as users of the technology produced some impressive successes in a short time period, followed by significant expansion in the COVID-19 (coronavirus disease 2019) context.

#### Co-design as a separate (prior) phase—or as ongoing practice

There were further tensions in how projects managed co-design, either as a bounded episodic activity preceding technology implementation, or as extended and emergent practice across different stages of service development. For example, in the epilepsy case, an affordable off-the-shelf activity tracker had been chosen, not only to provide a sense of normalcy for patients (so that the device would not be seen as stigmatizing), but also because an established product was assumed to be more reliable and technically able to extract necessary sensor data. It soon became apparent, however, that the head start gained by using an established product, made subsequent co-design challenging:

*“[Using established technology] means that you get up and running relatively quickly. When you're then trying to optimise your solution […] you're then struggling against bits of technology that you don't have real control or influence over.”* (Epilepsy case, interview 22, consultant neurologist)

The heart failure case also focused on an explicit, bounded technology co-design phase before the trial began. Adaptations continued as part of the trial, including invisible (and, sometimes, off-protocol) work undertaken by research nurses to address patients’ network connection problems (and linked technical work to enable both 3G and wifi transmission) or to strengthen care pathways (eg, collecting data from hospital records, making phone calls to confirm medication changes); however, these were mostly seen as quick fixes in the context of the study, rather than opportunities to co-shape the technology-supported service in a way that created shared values and vision:

*“We've had a lot of problems, like patients have connectivity and they seem alright and then we get back to the office and we just don’t get any readings, and then we have to sort of try and work out what's wrong.”* (Interview 1, trial team)

The video consultations project found that it was easier to engage patients (and to a certain extent also clinicians) in co-design when there was something tangible to talk about in the context of the service, rather than in the abstract:

*“We actually found it difficult to engage people on the general concept of ‘come and co-design with me’ but we found it very straightforward to engage on the ‘you’ve got an appointment, do you mind having that appointment [on video] and testing it out with us.’”* (Video consultations case, interview 1b, project lead)

Co-design was seen as an ongoing, practical activity constantly seeking to involve different direct and indirect (ie, those affected by the technology without using it directly) users. Significant visible and invisible work was carried out to feed into sustainable improvements, so that repetitive fixes to the same problems would not be needed.

All 3 projects recognized (to different degrees) that technology design was not an isolated process, but rather was closely coupled with clinical care, in which different kinds of expertise, experiential, clinical, and technological, came into dialogue and exchange:

*“It's difficult because you need somebody that has an understanding of the patients as well, because sometimes patients might then say to [the project manager from the commercial partner], ‘oh yeah, my app’s not working—this, this, and this—and I had a really bad seizure last night, and fell and hit my head.’ Well clinically, I need to know about the seizure, falling and banging the head.”* (Epilepsy case, interview 21, specialist nurse)

These findings point to the need for better recognition of how co-design can be facilitated, through the use of technologies that can accommodate necessary flexibility and through ongoing contribution of different kinds of expertise, so that added value will continue to be generated and sustained in sociotechnical practices. Beyond an abstract or artificial exercise, co-design could provide a mechanism for taking in account unpredictability and emergent change within service development. This also casts users as shaping the technology-supported service, rather than as passive adopters.

#### Infrastructures supporting extended co-design

Extended co-design has the potential to support the interdependent nature of technology development and service redesign. However, this seemed difficult to pursue without support from wider infrastructures (ie, institutional, regulatory and cultural, rather than purely technical),^42^ which often favored a narrow, technology-focused approach. Two of the projects, the pilot study in epilepsy and the heart failure RCT, partly depended on external research funding (at least in early stages) and were therefore attempting to prioritize requirements of funding calls. To contain and minimize risk, it often appeared easier to focus on immediate gains for the technology rather than attempt to engage with the complexity of technology-supported change in the National Health Service (NHS). Emergent design seemed a difficult fit with funding requirements:

*“And you know, complexity within the NHS is such that you probably need to think about it a bit more from first principles […] So, bringing together all of the necessary people and expertise, working out what is a reasonable plan, getting together sort of you know, kind of the logistics for it and the governance arrangements, and then put that in to a funding call is quite difficult.”* (Epilepsy case, interview 22, consultant neurologist)

In the example of the heart failure RCT, putting forward a case for an adaptive approach to technology development, in the context of this study design, was not straightforward. Implementing an ambitious vision for more efficient service provision also required significant work; however, limited resources meant only certain changes could be implemented:

*“The biggest challenge that we have in the study is the lack of resources […] implementing the vision we have is a huge work, and we have one developer here who is just a genius really; he's absolutely impressive but even he cannot do it as quickly.”* (Heart failure case, interview 4, project manager)

Lack of organizational resources and path dependency with legacy systems also hindered the potential for implementing necessary changes in epilepsy (regardless of whether they resulted from co-design or other processes):

*“The problem is that [the hospital] doesn't have any money to spend on the upgrade to the latest version of EPR. So, we have a funding problem. The code that the current version of EPR, which is 2 versions behind the current version, sits on, doesn't allow us to have that kind of flexibility.”* (Epilepsy case, interview 22, consultant neurologist)

In the video consultations case study, service reconfiguration and on-going co-design became easier due to significant political support for the project. This meant that human resources with the right expertise were available (eg, dedicated quality improvement lead, IT systems engineer) and had the right leverage not only to be able to request input from a wide range of clinical and nonclinical staff within the service, but also to successfully implement infrastructural changes resulting from fluid and ongoing co-design (eg, changes to space arrangements, availability of equipment). The relative success of this initiative to date (though it is still early days) may be partly attributable to the strong ethos in Scotland of the NHS as a public good, with a focus on reducing inequalities (specifically, addressing poor access to services for rural and remote patients).

## DISCUSSION

Our empirical findings illustrate that, although necessary, technical, mechanistic co-design is not sufficient for mainstreaming technology in health care. Instead, emphasis on the ongoing, social co-production of technology-supported services may allow better engagement with the tensions that surface in complex technology projects. A preliminary co-design “phase” helps the technology get to a reasonably mature stage, but iterative, adaptive co-design of the technology in “perpetual beta” mode may enable it to become sustainably embedded in the wider service.

Reorienting our focus toward technology-supported services also implies a shift from co-designing with technology users to co-designing with patients as service users, and with healthcare staff as professionals. Health technologies create new forms of knowledge and new possibilities for care that place (sometimes hidden) burdens on patients and carers, and require fundamental changes to staff roles and service models.^43^ Negotiating underpinning values and standards built into new models of technology-supported care, as well as engaging staff and patients in co-shaping pathways, routines and shared visions, becomes important.^43^ As Swinglehurst^44^ suggested, health technologies become enmeshed in the display and circulation of authority in clinical consultations, legitimizing particular ideals of what good care consists of. Distancing key groups from co-design may lead to disengagement from the articulations of the technology and divergence from work practices that could affect the success of technology projects. Instead, resilient organizations would support and encourage staff to continually bridge the gap between work-as-imagined and work-as-done, as well as feeding back into system learning and the evolution and adaptation of work routines and practices, to make health care safer.^45^

Drawing on our cross-case learning on service, rather than technology, co-design, we have devised the following principles: (1) co-design needs to be anchored in articulated and emerging needs of both direct and indirect users, and of those who will be affected by the technology in different ways; (2) ongoing co-design in complex systems does not mean unplanned and haphazard efforts, but rather requires adaptive capability to recognize emergence and manage unpredictability; (3) healthcare staff need support to accommodate ongoing co-design of a “perpetual beta” mode for technology in the service (so that when technical glitches happen, they are not perceived as unprofessional on their part); and (4) adequate resourcing and infrastructures need to be in place to enable co-design to reach its full potential.

Co-production of technology-supported services (rather than just co-designing patient-facing technical components) needs to embrace a more “open” approach, rather than operating around a predefined problem specification and largely fixed solution. We are not suggesting there is one “correct” approach or road map, nor do we believe that co-design will necessarily lead to a successful project. Rather, we propose that co-design can encourage a more reflective view, recognizing what the different trade-offs mean for technology-supported service change, and the crucial role which institutional and system capacity play in sustainably embedding technologies.^42^

### Strengths and limitations

This study compares and contrasts 3 different case studies of technology-supported change in the UK NHS (1 regional and 2 national). We have drawn on a large dataset to generate an in-depth understanding of how the cases evolved. However, we were not able to directly observe all co-design activities, as many took place prior to our involvement, though our dataset included contemporaneous communications, in-depth interviews with those involved, and published accounts of early-phase activities. While we sought a maximum variation sample of interviewees with different experiences with the technologies, those with negative views may be underrepresented. Despite the UK focus, we believe that our findings have broader implications, and our recommendations can be applied internationally.

### Implications for further research and practice

Complex, multilevel change requires ecological and sociotechnical perspectives to supplement mechanical efforts in introducing or mainstreaming innovations.^46^ More in-depth, detailed descriptions of co-design efforts are needed, using ethnographic approaches, to learn how to balance tensions between early co-design phases and ongoing, iterative adaptations. To achieve ongoing co-design in practice, organizational and technical infrastructures need to be upgraded. For example, hospital IT departments could play a leading role in facilitating innovation, rather than assuming a secondary support function.

## CONCLUSION

To bring back the social in sociotechnical co-design, we need to play closer attention to co-designing technology-supported services, instead of focusing too narrowly on technology development as an end in itself. This involves actively managing tensions around which groups to engage in service co-design, how to do so and to what extent; how to link early phase technology co-design with subsequent ongoing adaptations in practice; and being able to draw on institutional and organizational infrastructures to accomplish co-designed changes. Technologies that co-evolve alongside work practices and organizational routines have the potential to become better embedded in the health service and patient self-care.

## Ethical approvals

Case 1 received ethical approval by the London - Harrow Research Ethics Committee (REC no. 17/LO/1731). Ethical approval for case 2 was obtained in September 2015 from Oxfordshire South Central Research Ethics Committee (REC no. 15/SC/0553) and subsequent amendments. For case 3, approval was obtained from the UK Health Research Authority in June 2019 (REC no. 19/LO/0550) and linked permissions from local sites across Scotland. Interviews were audio-recorded with participant consent.

## FUNDING

Our studies on cases 1 and 2 are part of the Studies in Co-creating Assisted Living Solutions programme supported by a Senior Investigator Award to TG from the Wellcome Trust in its Society and Ethics Programme (WT104830MA). For case 1, CP also received an Academy of Medical Sciences Springboard Health of the Public 2040 award, funded by the Wellcome Trust in the United Kingdom (HOP001\1049). Case 3 is mainly funded by a research contract award from the Scottish Government, and co-design received additional funding from the Health Foundation.

## AUTTHOR CONTRIBUTIONS

The 3 case studies were conceptualized by TG, CP, JW and SS. CP, JW and TG were involved in data collection and analysis. CM led the co-design for case 3. CP and TG wrote the article; all other authors contributed to its refinement. All authors have seen and approved the final manuscript.

## Notes

Submission for consideration for the Special Focus Issue on the Continuing Legacy of Diana Forsythe’s Work at the Intersection of Informatics and the Social Sciences
